# Within amygdala: Basolateral parts are selectively impaired in premature-born adults

**DOI:** 10.1016/j.nicl.2021.102780

**Published:** 2021-08-09

**Authors:** Benita Schmitz-Koep, Juliana Zimmermann, Aurore Menegaux, Rachel Nuttall, Josef G. Bäuml, Sebastian C. Schneider, Marcel Daamen, Henning Boecker, Claus Zimmer, Dieter Wolke, Peter Bartmann, Dennis M. Hedderich, Christian Sorg

**Affiliations:** a Department of Diagnostic and Interventional Neuroradiology, School of Medicine, Technical University of Munich, Ismaninger Str. 22, Munich 81675, Germany; b TUM-NIC Neuroimaging Center, School of Medicine, Technical University of Munich, Ismaninger Str. 22, Munich 81675, Germany; c Functional Neuroimaging Group, Department of Diagnostic and Interventional Radiology, University Hospital Bonn, Venusberg-Campus 1, Bonn, Germany; d Department of Neonatology, University Hospital Bonn, Venusberg-Campus 1, Bonn, Germany; e Department of Psychology, University of Warwick, University Road, Coventry CV4 7AL, United Kingdom; f Warwick Medical School, University of Warwick, University Road, Coventry CV4 7AL, United Kingdom; g Department of Psychiatry, School of Medicine, Technical University of Munich, Ismaninger Str. 22, Munich 81675, Germany

**Keywords:** Premature birth, Human, Brain development, Amygdala nuclei, Basolateral amygdala, Structural magnetic resonance imaging, Social anxiety, AAA, anterior amygdaloid area, AB, accessory basal nucleus, Ba, basal nucleus, BLA, basolateral amygdala, BLS, Bavarian longitudinal study, BW, birth weight, CAT, corticoamygdaloid transition area, Ce, central nucleus, CI, confidence interval, CMA, centromedial amygdala, Co, cortical nucleus, CNS, central nerve system, DSM, Diagnostic and Statistical Manual of Mental Disorders, FDR, false discovery rate, FT, full-term, GA, gestational age, GABA, gamma aminobutyric acid, IQ, intelligence quotient, La, lateral nucleus, Me, medial nucleus, MPRAGE, magnetization prepared rapid acquisition gradient echo, MRI, magnetic resonance imaging, PL, paralaminar nucleus, SE, standard error, SFA, superficial amygdala, SPN, subplate neuron, TE, echo time, TI, inversion time, TIV, total intracranial volume, TR, repetition time, VLBW, very low birth weight, VP, very preterm, VP/VLBW, very preterm and/or very low birth weight, YASR, Young Adult Self Report

## Abstract

While it is known that whole amygdala volume is lastingly reduced after premature birth, it is unknown whether different amygdala nuclei are distinctively affected by prematurity. This question is motivated by two points: First, the observation that developmental trajectories of superficial, centromedial and basolateral amygdala nuclei are different. And second, the expectation that these different developmental pathways are distinctively affected by prematurity. Furthermore, we stated the question whether alterations in amygdala nuclei are associated with increased adults’ anxiety traits after premature birth.

We investigated 101 very premature-born adults (<32 weeks of gestation and/or birth weight below 1500 g) and 108 full-term controls of a prospectively and longitudinally collected cohort at 26 years of age using automated amygdala nuclei segmentation based on structural MRI.

We found selectively reduced volumes of bilateral accessory basal nuclei (pertaining to the basolateral amygdala of claustral developmental trajectory) adjusted for whole amygdala volume. Volumes of bilateral accessory basal nuclei were positively associated with gestational age and negatively associated with duration of ventilation. Furthermore, structural covariance within the basolateral amygdala was increased in premature-born adults. We did not find an association between reduced volumes of basolateral amygdala and increased social anxiety in the prematurity group.

These results demonstrate specifically altered basolateral amygdala structure in premature-born adults. Data suggest that prematurity has distinct effects on amygdala nuclei.

## Highlights

• Amygdala volume is reduced in premature-born adults.
• Particularly accessory basal nuclei volumes are selectively reduced.
• Structural covariance within basolateral amygdala is altered after premature birth.
• Data suggest that prematurity has lasting and distinct effects on amygdala nuclei.
• Basolateral amygdala development seems to be specifically impaired.

## 1 Introduction

Very premature birth (i.e., <32 weeks of gestation and/or birth weight <1500 g) affects multiple brain systems. These include the cerebral cortex, e.g. cortical microstructure and gyrification, the brain’s white matter, e.g. cortico-thalamic structural connectivity, and subcortical grey matter structures, e.g. multi-nuclei structures such as thalamus and neuromodulatory nuclei such as basal cholinergic forebrain, with most of these structural changes being linked with adverse neurodevelopmental outcomes (Ball et al., 2013, Berndt et al., 2019, Eikenes et al., 2011, Grothe et al., 2017, Hedderich et al., 2019, Hedderich et al., 2020a, Hedderich et al., 2020b, Menegaux et al., 2020, Menegaux et al., 2021, Meng et al., 2016, Northam et al., 2011, Nosarti et al., 2002, Nosarti et al., 2008, Pascoe et al., 2019, Rimol et al., 2019, Schmitz‐Koep et al., 2020, Skranes et al., 2007, Skranes et al., 2013). One subcortical grey matter structure of particular interest is the amygdala, which is thought to mediate the brain’s stress response to environmental stressors in the context of premature birth (Chau et al., 2019, Scheinost et al., 2016, Schmitz-Koep et al., 2021). Prematurely born neonates and infants are exposed to many potential stressors such as maternal separation, extra-uterine conditions under conditions of immaturity, pain, diagnostic procedures, and treatment procedures such as mechanical ventilation and potential surgery, which have been associated with altered brain development (Anand, 2000, Duerden et al., 2018, Grunau, 2013, Ranger et al., 2013, Smith et al., 2011). Stress exposure in turn modulates amygdala structure and function, making it vulnerable to prematurity (Hölzel et al., 2010, McEwen et al., 2016, Roozendaal et al., 2009). Indeed, reduced whole amygdala volumes have already been described in neonates, children, and adults after premature birth (Cismaru et al., 2016, Peterson et al., 2000, Schmitz-Koep et al., 2021), indicating lasting changes in amygdala structure in human prematurity.

**Fig. 1:**
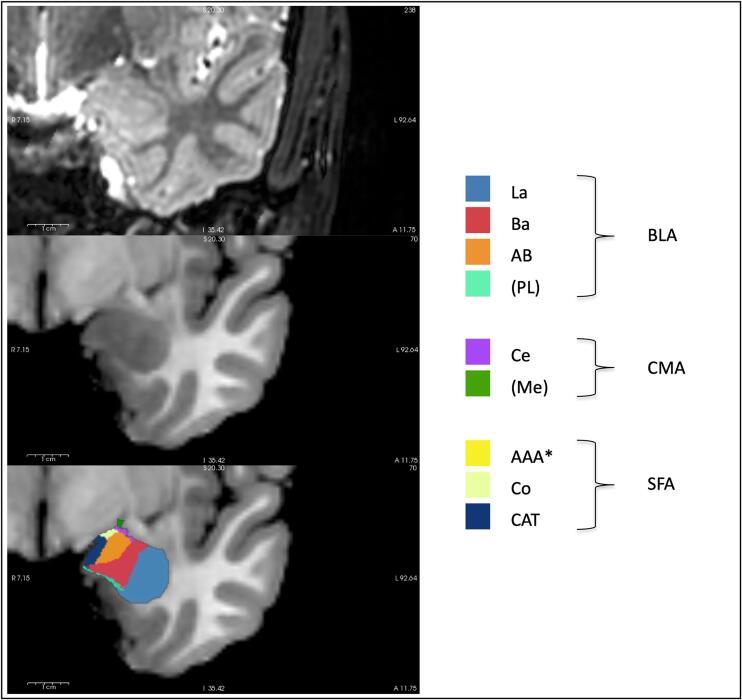
**Segmentation of amygdala nuclei.** T2-weighted and T1-weighted images of the amygdala and amygdala nuclei as segmented by FreeSurfer. *Not illustrated. Abbreviations: AAA, Anterior amygdaloid area; AB, accessory basal nucleus; Ba, basal nucleus; BLA, basolateral amygdala; CAT, corticoamygdaloid transition area; Ce, central nucleus; CMA, centromedial amygdala; Co, cortical nucleus; La, lateral nucleus; Me, medial nucleus; PL, paralaminar nucleus; SFA, superficial amygdala.

However, the amygdala is not a homogenous structure. Instead, it consists of several grey matter nuclei which are typically divided into three groups based on their distinct developmental pathways (Pape and Pare, 2010, Sah et al., 2003, Swanson and Petrovich, 1998): First, a superficial division (SFA) which is part of the cortex with a corresponding cortical developmental trajectory for particular glutamatergic neurons, second, a centromedial division (CMA) which is thought of as a ventromedial expanse of the striatum with a corresponding striatal developmental trajectory for particular GABAergic neurons, and third, a basolateral division (BLA) which is derived from a ventromedial extension of claustrum anlage with a corresponding claustral developmental trajectory for particular subplate and glutamatergic neurons (Bruguier et al., 2020, Medina et al., 2004, Pape and Pare, 2010, Puelles, 2014, Sah et al., 2003, Swanson and Petrovich, 1998, Watson and Puelles, 2017). These three groups within the amygdala are also distinct based on cytoarchitectonic mapping, with different dominating cell types within each group (Amunts et al., 2005, Heimer et al., 1999): For example, while BLA contains mainly glutamatergic projection cells and secondly local-circuit GABAergic cells, CMA mainly consists of GABAergic cells similar to striatal neurons (McDonald, 1982, Pape and Pare, 2010, Sah et al., 2003). Critical for the current study is that advances in automated brain segmentation have made it possible to identify distinct amygdala nuclei by in-vivo structural MRI in humans (Saygin et al., 2017), facilitating nuclei sensitive analysis of the adult amygdala. The assignment of each nucleus to SFA, CMA or BLA is illustrated in Fig. 1. Furthermore, it is important for our approach that these different, superficial, centromedial, and basolateral, nuclei groups are associated with distinct, cortical, striatal, and claustral developmental trajectories, as described above (Amunts et al., 2005, Pape and Pare, 2010, Sah et al., 2003, Swanson and Petrovich, 1998), which might in turn reflect distinct vulnerability to impaired development in prematurity. For example, perinatal adverse events, such as transient hypoxia/ischemia, particularly affect subplate neurons (SPNs), which are distinctively involved in claustrum and thereby BLA development (McClendon et al., 2017). It is still unknown, however, whether these developmental trajectories might lead to differential effects of prematurity on the individual amygdala nuclei in humans.

Furthermore, premature birth is associated with an increased risk for social impairments (Eryigit-Madzwamuse et al., 2015, Johnson, 2007, Pesonen et al., 2008, Wolke et al., 2019). Accordingly, we recently found significantly higher scores on an avoidant personality scale in a cohort of premature-born young adults, reflecting increased social anxiety trait (Schmitz-Koep et al., 2021). Still, although the amygdala has been linked with social anxiety in general and although social impairments were associated with altered functional connectivity of the amygdala in preterm-born adolescents (Davidson, 2002, Davis and Whalen, 2001, Johns et al., 2019), we did not find evidence that social anxiety was correlated with global amygdala volume alterations (Schmitz-Koep et al., 2021). However, as mentioned above, the amygdala is not a homogenous structure but consists of several nuclei, which can be assigned to three subdivisions based on their distinct developmental pathways. Within the amygdala, functional specialization and parallel processing take place (Balleine and Killcross, 2006, Janak and Tye, 2015). For example, evidence regarding differential roles of amygdala subdivisions in humans comes from studies investigating Urbach-Wiethe patients (Hortensius et al., 2017). These studies suggest deficits in the processing of ambiguous social information, and impaired learning from social experience in humans with BLA damage (de Gelder et al., 2014, Rosenberger et al., 2019). It remains unclear, whether distinct amygdala nuclei might mediate social impairments in premature-born adults.

Following up on previous work (Schmitz-Koep et al., 2021), in the present study we investigated 101 very premature-born adults and 108 full-term controls at 26 years of age using automated FreeSurfer segmentation of amygdala nuclei (Saygin et al., 2017) in structural MRI to address the following questions: First, are there differential effects of prematurity on individual amygdala nuclei structure? As proxy for amygdala nuclei structure, we used both nuclei volume and structural covariance among nuclei. And second, are structural differences in distinct amygdala nuclei linked with social anxiety?

## 2 Methods

### 2.1 Participants

Our study sample was previously described in (Riegel et al., 1995, Schmitz-Koep et al., 2021, Wolke et al., 1994, Wolke and Meyer, 1999): All subjects were part of the Bavarian Longitudinal Study (BLS), a geographically defined, whole-population sample of neonatal at-risk children and healthy FT controls who were followed from birth, between January 1985 and March 1986, into adulthood (Eryigit Madzwamuse et al., 2015, Reyes et al., 2021). 682 infants were born VP (<32 weeks of gestation) and/or with very low birth weight (VLBW, birth weight <1500 g). Informed consent from a parent and/or legal guardian was obtained. From the initial 916 FT born infants born at the same obstetric hospitals that were alive at 6 years, 350 were randomly selected as control subjects within the stratification variables of sex and family socioeconomic status in order to be comparable with the VP/VLBW sample. Of these, 411 VP/VLBW individuals and 308 controls were alive and eligible for the 26-year follow-up assessment. 260 from the VP/VLBW group and 229 controls participated in psychological assessments (Breeman et al., 2015). All subjects were screened for MR-related exclusion criteria including (self-reported): claustrophobia, inability to lie still for >30 min, unstable medical conditions (e.g. severe asthma), epilepsy, tinnitus, pregnancy, non-removable MRI-incompatible metal implants and a history of severe CNS trauma or disease that would impair further analysis of the data. However, the most frequent reason not to perform the MRI exam was that subjects declined to participate. Finally, 101 VP/VLBW subjects and 111 FT controls underwent MRI at 26 years of age. The MRI examinations took place at two sites: The Department of Neuroradiology, Klinikum rechts der Isar, Technische Universität München, (n = 145) and the Department of Radiology, University Hospital of Bonn (n = 67). The study was carried out in accordance with the Declaration of Helsinki and was approved by the local ethics committee of the Klinikum rechts der Isar, Technische Universität München and the University Hospital Bonn. All study participants gave written informed consent. They received travel expenses and a small payment for participation.

### 2.2 Birth variables

Gestational age (GA) in weeks was estimated from maternal reports on the first day of the last menstrual period and serial ultrasounds during pregnancy. In cases in which the two measures differed by more than 2 weeks, clinical assessment at birth with the Dubowitz method was applied (Dubowitz et al., 1970). Birth weight (BW) in grams was obtained from obstetric records. Duration of mechanical ventilation in days was computed from daily records by research nurses.

### 2.3 Variables related to anxiety

To assess behavioral and emotional outcome related to anxiety, we used the German version of the Young Adult Self Report (YASR) which includes six (Depressive, Anxiety, Somatic, Avoidant personality, Attention deficit/hyperactivity problems, and Antisocial personality) included DSM-IV-oriented scales (Achenbach, 1997). In a previous study of the present cohort, we found significantly higher T score for avoidant personality in VP/VLBW individuals compared to FT controls, indicating increased social anxiety trait (Schmitz-Koep et al., 2021). Therefore, we chose the avoidant personality score for these analyses.

### 2.4 MRI data acquisition

MRI data acquisition was described previously (Hedderich et al., 2020a, Schmitz-Koep et al., 2021): At both sites, Bonn and Munich, MRI data acquisition was performed on Philips Achieva 3 T TX systems or Philips Ingenia 3 T system using an 8-channel SENSE head coil (subject distribution among scanners: Bonn Achieva 3 T: 5 VP/VLBW, 12 FT, Bonn Ingenia 3 T: 33 VP/VLBW, 17 FT, Munich Achieva 3 T: 60 VP/VLBW, 65 FT, Munich Ingenia 3 T: 3 VP/VLBW, 17 FT). To account for possible confounds by scanner differences, data analyses included scanner dummy-variables as covariates of no interest. Across all scanners, sequence parameters were kept identical. Scanners were checked regularly to provide optimal scanning conditions and MRI physicists at the University Hospital Bonn and Klinikum rechts der Isar regularly scanned imaging phantoms, to ensure within-scanner signal stability over time. Signal-to-noise ratio was not significantly different between scanners (one-way ANOVA with factor ‘scanner-ID’ [Bonn 1, Bonn 2, Munich 1, Munich 2]; F(3,182) = 1.84, p = 0.11). A high-resolution T1-weighted 3D-MPRAGE sequence (TI = 1300 ms, TR = 7.7 ms, TE = 3.9 ms, flip angle = 15°, field of view = 256 mm × 256 mm, reconstruction matrix = 256 × 256; reconstructed isotropic voxel size = 1 mm^3^) and a high resolution T2-weighted 3D sequence (TR = 2500 ms, TE = 364 ms, flip angle = 90°; field of view = 512 mm × 512 mm, echo train length = 120, reconstructed isotropic voxel size = 0.5 mm^3^) were acquired. All images were visually inspected for artifacts.

### 2.5 MRI processing and amygdala segmentation

Images saved as DICOMs were converted to Nifti-format using dcm2nii (Li et al., 2016). The FreeSurfer image analysis suite, version 6.0 (http://surfer.nmr.mgh.harvard.edu/) was used which includes an automated segmentation of the amygdala nuclei (Saygin et al., 2017). Recently, Armio et al. (Armio et al., 2020) used this algorithm to investigate amygdala subnucleus volumes in psychosis high-risk state and first-episode psychosis. They assessed reliability of the segmentation method scanning five subjects twice and showed excellent test-retest reliability (Armio et al., 2020). Using both high-resolution T1-weighted and T2-weighted images, nine amygdala nuclei were labeled per hemisphere: Anterior amygdaloid area (AAA), corticoamygdaloid transition area (CAT), basal nucleus (Ba), lateral nucleus (La), accessory basal nucleus (AB), central nucleus (Ce), cortical nucleus (Co), medial nucleus (Me) and paralaminar nucleus (PL). Segmentation outputs were inspected visually. Examples of amygdala segmentation are shown in Fig. 1. Successful amygdala segmentations were available in 101 VP/VLBW subjects and 109 FT subjects. These nine amygdala nuclei were assigned to one of the three amygdala subdivisions as visualized in Fig. 1. SFA includes AAA, CAT and Co, CMA includes Ce and Me, and BLA includes Ba, La, AB and PL.

However, segmentation of amygdala nuclei is challenging due to small regional volumes and limited availability of a clear ground truth. Buser et al. (Buser et al., 2020) assessed the spatial and numerical reliability for the segmentation of amygdala nuclei in FreeSurfer. While numerical reliability was mostly high within the amygdala, medial nucleus and paralaminar nucleus showed poor spatial reliability (Buser et al., 2020). Therefore, we decided to exclude both medial and paralaminar nucleus from our analyses.

Estimation of total intracranial volume (TIV) was performed with the CAT12 toolbox, version r1364 (http://www.neuro.uni-jena.de/cat/) (Gaser and Dahnke, 2016) within SPM12 (https://www.fil.ion.ucl.ac.uk/spm/software/spm12/).

### 2.6 Statistical analysis

All statistical analyses were performed using IBM SPSS Version 26 (IBM Corp., Armonk, NY, USA). To detect possible outliers, we used a method proposed by Hoaglin and Iglewicz (1987) which multiplies the interquartile range by the factor 2.2 to determine outliers. One FT subject was excluded from the analyses because it contained multiple outlier values. Finally, 101 VP/VLBW subjects and 108 FT subjects were included in the analyses. Age was not included as a covariate in our analyses, as VP/VLBW subjects and FT controls had the same mean age of 26 years (p = 0.165).

### 2.7 Outcome measures

#### 2.7.1 Amygdala nuclei volumes after premature birth

Our first outcome measure of amygdala nuclei structure was nuclei volume. To test whether specific nuclei of the amygdala are particularly affected by alterations in volume after premature birth, we used general linear models. We performed 14 separate tests entering the respective amygdala nucleus (left La, left Ba, left AB, left AAA, left Ce, left Co, and left CAT, as well as these volumes in the right hemisphere) as dependent variables, group membership as fixed factor and whole amygdala volume of the left and right hemisphere, respectively, sex and scanner as covariates.

We conducted two control analyses: First, to investigate the effect of adjusting for whole amygdala volume, we repeated general linear model analyses with TIV, sex and scanner, not left or right whole amygdala volume, as covariates.

Second, there were few subjects with intraventricular hemorrhage in the neonatal period (see Table S4). To investigate whether removing these subjects impacts the results, we repeated general linear model analyses for left and right accessory basal nucleus between the remaining subjects of the VP/VLBW group and the FT group. We entered volumes of left and right accessory basal nucleus as dependent variables, group membership as fixed factor and whole amygdala volume, sex and scanner as covariates.

To test whether differences in amygdala nuclei volumes between VP/VLBW subjects and FT controls are specifically related to premature birth, we conducted a two-tailed partial correlation analysis in the VP/VLBW group. If group differences were found, then nuclei volumes were correlated with GA, BW and duration of ventilation as variables of premature birth. TIV, sex and scanner were entered as covariates.

#### 2.7.2 Amygdala nuclei structural covariance

Our second outcome measure of amygdala nuclei structure was structural covariance. It has been proposed that linked brain regions may develop in concert, and that coordinated development, for example of cortical regions, is altered after premature birth. Therefore, we investigated interrelated development within the amygdala, i.e., structural covariance (Alexander-Bloch et al., 2013, Nosarti et al., 2011, Scheinost et al., 2017). To explore structural covariance within the amygdala, we tested the correlation between amygdala nuclei volumes in the VP/VLBW and FT group. Since we found reduced volumes of accessory basal nuclei in premature-born adults, we focused our structural covariance analysis on these nuclei. More specifically, we entered volumes of the left and right accessory basal nucleus and ipsilateral amygdala nuclei, respectively, as variables of interest into a two-tailed partial correlation analysis in each group (VP/VLBW and FT) separately. TIV, sex and scanner were entered as covariates of no interest. We tested for differences in structural covariance between VP/VLBW subjects and FT controls using Fisher r-to-z transformation and calculating z-scores and p-values to assess the significance of the difference (Eid et al., 2013).

#### 2.7.3 Thresholding and correction for multiple testing

All analyses were FDR corrected for multiple comparisons using the Benjamini-Hochberg procedure (Benjamini and Hochberg, 1995). Statistical significance was defined as p < 0.05.

### 2.8 Correlation between amygdala nuclei volumes and anxiety

We used the avoidant personality T scores to study the relationship between altered amygdala nuclei volumes and social anxiety. In the VP/VLBW group, we entered amygdala nuclei volumes (i.e., left and right accessory basal nucleus volumes), respectively, and anxiety scores (i.e., the avoidant personality T scores) as variables of interest and TIV, sex and scanner as covariates into a two-tailed partial correlation analysis. Results were FDR corrected for multiple comparisons using the Benjamini-Hochberg procedure (Benjamini and Hochberg, 1995). Statistical significance was defined as p < 0.05.

### 2.9 Data availability statement

Patient data used in this study are not publicly available but stored by the principal investigators of the Bavarian Longitudinal Study.

## 3 Results

### 3.1 Sample characteristics

Demographic and clinical background variables are presented in Table 1. Sex (p = 0.894) and age at scanning (p = 0.165) did not differ significantly between the VP/VLBW group and the FT group. By design of the study, GA (p < 0.001) and BW (p < 0.001) were significantly lower in the VP/VLBW group compared to FT controls. Furthermore, TIV was significantly smaller in VP/VLBW individuals compared to FT controls (p = 0.001).

**Table 1:** Demographical, clinical and cognitive data.

	VP/VLBW (n = 101)			FT (n = 108)			p-value
	Mean	SD	Range	Mean	SD	Range	
Sex (male/female)	58/43			63/45			0.894
Age (years)	26.7	± 0.61	25.7 – 28.3	26.8	± 0.75	25.5 – 28.9	0.165
GA (weeks)	30.5	± 2.1	25 – 36	39.7	± 1.1	37 – 42	**<0.001**
BW (g)	1325	± 313	630 – 2070	3389	± 447	2120 – 4670	**<0.001**
Ventilation (days)	12.1	± 17.5	0 – 81	n.a.	n.a.	n.a.	n.a.
TIV (mm^3^)	1457.9	± 141.1	1132.3 – 1793.6	1524.0	± 146.1	1166.2 – 1918.7	**0.001**

YASR DSM-oriented scale, T score	Mean	SE	Range	Mean	SE	Range	p-value
Anxiety	52.0	± 4.1	50 – 70	51.2	± 2.9	50 – 67	0.097
Avoidant personality	55.5	± 8.0	50 – 83	52.5	± 4.9	50 – 73	**0.001**

Statistical comparisons: sex with χ^2^ statistics; age, GA, BW, TIV and YASR with two sample t-tests. Bold letters indicate statistical significance defined as p < 0.05.

Abbreviations: BW, birth weight; DSM, Diagnostic and Statistical Manual of Mental Disorders; FT, full-term; GA, gestational age; SD, standard deviation; SE, standard error; VP/VLBW, very preterm and/or very low birthweight, YASR, Young Adult Self Report.

### 3.2 Altered structure of basolateral amygdala in premature-born adults

**Fig. 2:**
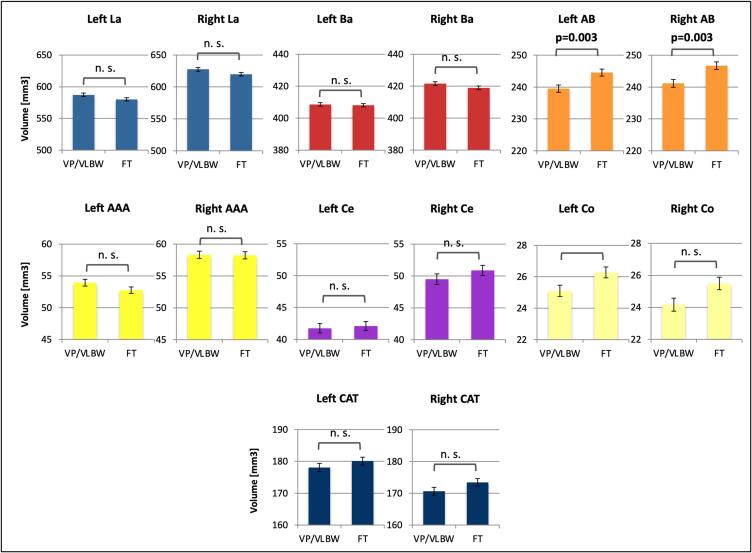
**Amygdala nuclei volumes – group comparison.** Estimated marginal means of amygdala nuclei volumes (in mm^3^) are shown as bar charts with SE (in mm^3^) as error bars. Bold letters indicate statistical significance after FDR correction using the Benjamini-Hochberg procedure. Abbreviations: AAA, Anterior amygdaloid area; AB, accessory basal nucleus; Ba, basal nucleus; CAT, corticoamygdaloid transition area; Ce, central nucleus; Co, cortical nucleus; FT, full term; La, lateral nucleus; n. s., not significant; SE, standard error; VP/VLWB, very preterm and/or very low birth weight.

Automated segmentation of the amygdala nuclei in structural MRI data is visualized in Fig. 1. To investigate whether specific nuclei of the amygdala are particularly affected by alterations in volume after premature birth, we used general linear models. After FDR correction for multiple comparisons, both left and right accessory basal nucleus showed significantly lower volume in VP/VLBW subjects compared to controls. Fig. 2 and Table 2 present estimated marginal means and p-values. Table S1 presents raw amygdala nuclei volumes.

**Table 2:** Group comparison of amygdala nuclei volumes.

	VP/VLBW (n=101)				FT (n=108)				p-value
	mean	SE	95% CI		mean	SE	95% CI		
**Left**									
La	587.29	2.70	581.98	592.61	580.12	2.60	575.00	585.24	0.074
Ba	408.64	1.07	406.54	410.74	408.10	1.03	406.08	410.12	0.731
AB	239.50	1.14	237.25	241.75	244.54	1.10	242.37	246.70	**0.003**
AAA	53.93	0.53	52.88	54.98	52.74	0.51	51.73	53.75	0.134
Ce	41.75	0.74	40.29	43.20	42.10	0.71	40.70	43.50	0.747
Co	25.11	0.36	24.40	25.82	26.28	0.35	25.60	26.96	0.029
CAT	178.09	1.28	175.58	180.61	180.08	1.23	177.66	182.50	0.293

**Right**									
La	627.59	2.87	621.95	633.24	619.96	2.76	614.52	625.40	0.072
Ba	421.62	1.20	419.25	423.99	418.99	1.16	416.71	421.27	0.139
AB	241.17	1.23	238.74	243.59	246.72	1.19	244.39	249.06	**0.003**
AAA	58.32	0.58	57.17	59.47	58.24	0.56	57.13	59.34	0.925
Ce	49.50	0.83	47.85	51.14	50.86	0.80	49.28	52.45	0.267
Co	24.17	0.41	23.36	24.97	25.49	0.39	24.72	26.26	0.028
CAT	170.65	1.23	168.24	173.07	173.44	1.18	171.11	175.77	0.125

Estimated marginal means of amygdala nuclei volumes (in mm^3^) with SE (in mm^3^) and 95% CI (in mm^3^) in VP/VLBW subjects and in FT controls. General linear models with group membership as fixed factor and left or right whole amygdala volume, respectively, sex and scanner as covariates. Bold letters indicate statistical significance after FDR correction using the Benjamini-Hochberg procedure.

Abbreviations: AAA, anterior amygdaloid area; AB, accessory basal nucleus; Ba, basal nucleus; CAT, corticoamygdaloid transition area; Ce, central nucleus; CI, confidence interval; Co, cortical nucleus; FT, full term; La, lateral nucleus; SE, standard error; VP/VLWB, very preterm and/or very low birth weight.

We conducted two control analyses: First, to investigate the effect of adjusting for whole amygdala volume, we repeated general linear model analyses without left or right whole amygdala volume, but with TIV, sex and scanner as covariates. After FDR correction for multiple comparisons, all amygdala nuclei showed significantly lower volume in VP/VLBW subjects compared to FT controls. Table S2 presents estimated marginal means and p-values. The results indicate that while premature birth has an effect on all amygdala nuclei, accessory basal nucleus is particularly affected in relation to whole amygdala volume.

Second, in order to control for an impact of intraventricular hemorrhage on our results, we removed these subjects (see Table S4) and repeated general linear model analyses for accessory basal nucleus between the remaining subjects of the VP/VLBW group and the FT group. We found significantly lower volumes of left and right accessory basal nucleus in VP/VLBW subjects without intraventricular hemorrhage compared to FT controls (see Table S5), verifying that accessory basal nucleus – as part of BLA – seems to be particularly affected. These results indicate that our main findings of amygdala nuclei volume reductions were not affected by effects of intraventricular hemorrhage.

**Fig. 3:**
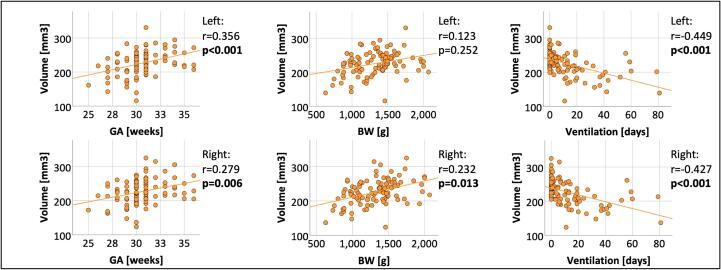
**Relationship between volume of the accessory basal nucleus and variables of premature birth.** The associations between left and right volume of accessory basal nucleus and GA, BW and duration of ventilation are shown as scatter plots. GA (in weeks), BW (in grams) and duration of ventilation (in days) are plotted on the x-axes. Volumes of accessory basal nucleus (in mm^3^) are plotted on the y-axes. Linear regression lines as well as correlation coefficients and p-values were added. Bold letters indicate statistical significance after FDR correction using the Benjamini-Hochberg procedure. Abbreviations: BW, birth weight; GA, gestational age.

To support the notion that volume reductions of both left and right accessory basal nucleus were specifically related to prematurity, we conducted a partial correlation analysis (Fig. 3, Table 3). We observed significant positive correlations between GA and volumes of both left (r = 0.356, p < 0.001) and right accessory basal nucleus (r = 0.279, p = 0.006). While there was no significant relationship between BW and left accessory basal nucleus volume (r = 0.123, p = 0.252), BW and right accessory basal nucleus volume showed a significant positive correlation (r = 0.232, p = 0.013). We found significant negative correlations between duration of ventilation and volumes of both left (r = -0.449, p < 0.001) and right accessory basal nucleus (r = −0.427, p < 0.001), possibly reflecting vulnerability of BLA to stress exposure induced by premature birth. Fig. 3 and Table 3 present correlation coefficients and p-values from the partial correlation analysis between volumes of accessory basal nuclei and variables of premature birth.

**Table 3:** Relationship between volumes of left and right accessory basal nucleus and variables of premature birth.

Risk factor	Volume	Correlation coefficient	p-value
GA	Left AB Right AB	0.356 0.279	**<0.001** **0.006**
BW	Left AB Right AB	0.123 0.232	0.252 **0.013**
Duration of Ventilation	Left AB Right AB	−0.449 –0.427	**<0.001** **<0.001**

Correlation coefficients and p-values from partial correlation analysis between the volume of the accessory basal nucleus and variables of premature birth. TIV, sex and scanner were entered as covariates. Bold letters indicate statistical significance after FDR correction using the Benjamini-Hochberg procedure.

Abbreviations: AB, accessory basal nucleus; BW, birth weight; GA, gestational age.

**Fig. 4:**
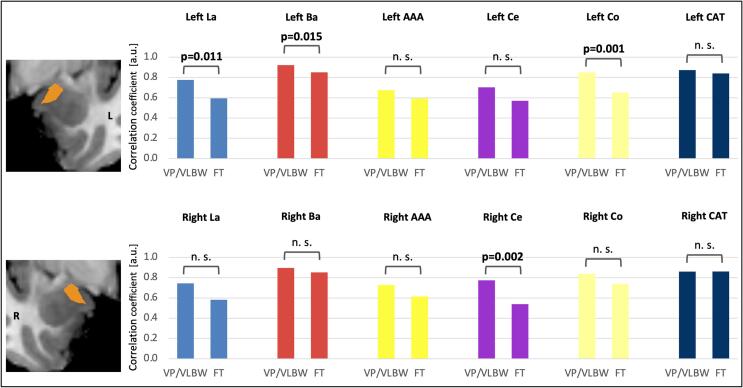
**Structural covariance analysis of accessory basal nucleus.** Correlation coefficients between left and right accessory basal nucleus and ipsilateral amygdala nuclei volumes, respectively, are shown as bar charts. Bold letters indicate statistical significance after FDR correction using the Benjamini-Hochberg procedure. Abbreviations: AAA, Anterior amygdaloid area; Ba, basal nucleus; CAT, corticoamygdaloid transition area; Ce, central nucleus; Co, cortical nucleus; FT, full term; L, left; La, lateral nucleus; n. s., not significant; R, right; VP/VLWB, very preterm and/or very low birth weight.

Furthermore, we investigated coordinated structural development within the amygdala using structural covariance. In order to explore structural covariance for the accessory basal nucleus, as this part of BLA was significantly smaller in premature-born adults, we tested the correlation between volumes of the left and right accessory basal nucleus and ipsilateral amygdala nuclei, respectively. Table 4 presents correlation coefficients from the partial correlation analyses, and p-values from comparing correlation coefficients between VP/VLBW subjects and FT controls. Fig. 4 visualizes group differences in correlation between amygdala nuclei volumes as bar charts. Expectedly, volumes of the accessory basal nucleus correlated highly with ipsilateral basal nucleus volumes, which is also part of BLA, in both groups (left hemisphere: VP/VLBW: r = 0.922, FT: r = 0.851; right hemisphere: VP/VLBW: r = 0.896, FT: r = 0.852), indicating concerted development within BLA. In the left hemisphere, the correlation was significantly higher in VP/VLBW individuals compared to FT controls (p = 0.015). Furthermore, correlation between volumes of the left accessory basal nucleus and left lateral nucleus (also part of BLA) was significantly higher in VP/VLBW individuals compared to FT controls (VP/VLBW: r = 0.777, FT: r = 0.593; p = 0.011). Moreover, correlation between volumes of the left accessory basal nucleus and left cortical nucleus (part of SFA) and correlation between volumes of the right accessory basal nucleus and right central nucleus (part of CMA) were significantly higher in VP/VLBW individuals compared to FT controls (VP/VLBW: r = 0.851, FT: r = 0.652; p = 0.001, and VP/VLBW: r = 0.774, FT: r = 0.539; p = 0.002, respectively).

**Table 4:** Correlation between amygdala nuclei volumes.

Volume	Volume	VP/VLBW (n = 101) Correlation coefficient	FT (n = 108) Correlation coefficient	z-score	p-value
**Left** AB	**Left** La Ba AAA Ce Co CAT	0.777 0.922 0.703 0.675 0.851 0.873	0.593 0.851 0.570 0.594 0.652 0.840	2.531 2.438 1.607 0.969 3.425 0.886	**0.011** **0.015** 0.108 0.333 **0.001** 0.376
**Right** AB	**Right** La Ba AAA Ce Co CAT	0.745 0.896 0.728 0.774 0.838 0.860	0.582 0.852 0.615 0.539 0.737 0.861	2.108 1.340 1.478 3.044 1.926 –0.027	0.035 0.180 0.140 **0.002** 0.054 0.978

Correlation coefficients from partial correlation analysis between volumes of left accessory basal nucleus and ipsilateral amygdala nuclei and volumes of right accessory basal nucleus and ipsilateral amygdala nuclei. TIV, sex and scanner were entered as covariates. Comparing correlations bold letters indicate statistical significance after FDR correction using the Benjamini-Hochberg procedure.

Abbreviations: AAA, anterior amygdaloid area; AB, accessory basal nucleus; Ba, basal nucleus; CAT, corticoamygdaloid transition area; Ce, central nucleus; Co, cortical nucleus; FT, full term; La, lateral nucleus; VP/VLWB, very preterm and/or very low birth weight.

In summary, our results showed that the accessory basal nucleus – as part of the basolateral subdivision – was significantly reduced in volume when whole amygdala volume was entered as a covariate. Furthermore, structural covariance between parts of left BLA was significantly higher in VP/VLBW individuals compared to FT controls. Hence, these results support the hypothesis that BLA is particularly affected by premature birth.

### 3.3 Increased social anxiety is not associated with reduced amygdala volumes in premature-born adults

**Fig. 5:**
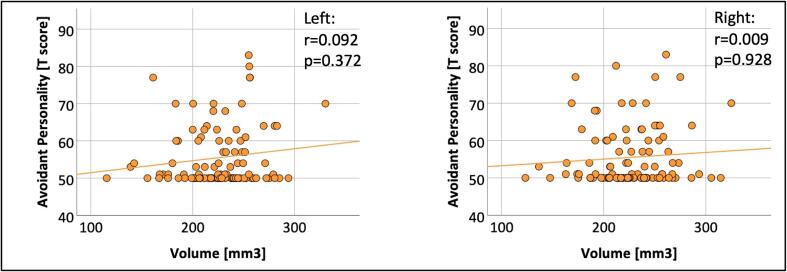
**Relationship between volume of the accessory basal nucleus and avoidant personality score.** The associations between left and right volume of accessory basal nucleus and the avoidant personality T score are shown as scatter plots. Volumes of accessory basal nucleus (in mm^3^) are plotted on the x-axes. The T score is plotted on the y-axes. Linear regression lines as well as correlation coefficients and p-values were added. Bold letters indicate statistical significance after FDR correction using the Benjamini-Hochberg procedure.

To investigate whether reduced amygdala nuclei volumes are linked with increased social anxiety in premature-born adults, we investigated the relationship between accessory basal nuclei volumes and the YASR avoidant personality score using a partial correlation analysis (Fig. 5, Table 5). There was no significant correlation between the T score for avoidant personality and left (r = 0.092, p = 0.372) or right accessory basal nucleus volume (r = 0.009, p = 0.928). Correlation coefficients and p-values are presented in Fig. 5 and Table 5.

**Table 5:** Relationship between volumes of left and right accessory basal nucleus and avoidant personality score.

YASR score	Volume	Correlation coefficient	p-value
Avoidant personality	Left AB Right AB	0.092 0.009	0.372 0.928

Correlation coefficients and p-values from partial correlation analysis between the volume of the accessory basal nucleus and the avoidant personality score. TIV, sex and scanner were entered as covariates. Bold letters indicate statistical significance after FDR correction using the Benjamini-Hochberg procedure.

Abbreviations: AB, accessory basal nucleus; CI, confidence interval; YASR, Young Adult Self Report.

These results suggest that, while part of the BLA is specifically reduced in volume after premature birth, there seems to be no association with social anxiety.

## 4 Discussion

Based on structural MRI, we demonstrated specifically reduced volumes of accessory basal nucleus – as part of BLA – and altered structural covariance within the amygdala in VP/VLBW subjects compared to FT controls at 26 years of age. There seems to be no association between these specific volumetric reductions and increased social anxiety. Results indicate, to the best of our knowledge for the first time, that prematurity affects subnuclei of amygdala specifically, namely BLA. Data suggest that BLA development is specifically impaired after premature birth, possibly due to disturbance of its distinct claustral developmental pathway.

### 4.1 Altered structure of basolateral amygdala after premature birth

All amygdala nuclei showed significantly lower volume in VP/VLBW subjects compared to FT controls, however, amygdala composition differed in VP/VLBW adults, since accessory basal nucleus showed significantly lower volumes adjusted for whole amygdala volume (Fig. 2). The accessory basal nucleus is one of four nuclei (together with basal, lateral and paralaminar nucleus) composing BLA. It integrates input from cortical and subcortical regions as well as from within the amygdala (for example lateral and basal nucleus) and projects to the central and medial nucleus as parts of autonomic pathways (Aggleton et al., 1980, Pitkänen et al., 1995, Sah et al., 2003, Savander et al., 1995). Previous studies suggested that BLA and claustrum, a thin sheet of grey matter between external and extreme capsule, both are of pallial origin (Medina et al., 2004, Waclaw et al., 2010). This is supported by evidence for presumably glutamatergic projection neurons in BLA similar to the cerebral cortex and the claustrum, and expression of a major glutamate transporter gene in the cerebral cortex which is also expressed both in BLA and claustrum (J. B. Smith et al., 2019, Swanson and Petrovich, 1998). In particular, BLA development may partly depend on subplate neuron (SPN) development: The subplate zone, mainly consisting of SPNs, is a largely transient structure that plays a particularly important role in the structural and functional organization of the cortex during its developmental peak between 22 and 34 weeks of gestation (Kostović et al., 1989, McConnell et al., 1989). A common developmental origin of subplate and claustrum has been proposed, as gene expression patterns suggest part of the claustrum to be subplate-like (Bruguier et al., 2020, Puelles, 2014, Watson and Puelles, 2017). As mentioned above, BLA is derived from a ventromedial extension of claustrum anlage. Hence, first, BLA is tightly linked to SPN development, and second, SPN damage is a key mediator of aberrant brain development after premature birth (McClendon et al., 2017). Therefore, BLA may be particularly vulnerable to disturbances in brain development after premature birth. However, the expression of a subplate-specific gene in the BLA was only observed in the mouse to date (Wang et al., 2010). Other potential explanations for BLA vulnerability after premature birth may include injury of other neurons involved in BLA, and especially accessory basal nucleus, development. However, the discussion of potential mechanisms behind BLA vulnerability has to be interpreted with care, since it is not clear whether findings reported from BLA can be generalized to all nuclei of BLA, including the accessory basal nucleus. Furthermore, since the accessory basal nucleus integrates input from cortical and subcortical regions, volume reductions may also be secondary to altered input projections. Lastly, amygdala volume reductions after premature birth have been associated with greater exposure to neonatal pain/stress (Chau et al., 2019), and in the present study we found significant negative correlations between duration of ventilation and volumes of both left and right accessory basal nucleus, possibly reflecting vulnerability to stress exposure induced by premature birth. Hence, volumetric differences could also stem from early injury or early differences related to prenatal, perinatal, and early postnatal stress, that remain in spite of superimposed later postnatal development.

Furthermore, it has been proposed that concerted development and connectivity of linked brain regions may be reflected by structural covariance (Alexander-Bloch et al., 2013, Lerch et al., 2006, Mechelli, 2005). After premature birth, increased as well as decreased covariance has been reported between cortical and subcortical regions and cerebellum in adolescents and young adults (Nosarti et al., 2011, Scheinost et al., 2017). More specifically, we previously investigated structural covariance of whole amygdala volume across hemispheres in premature-born adults (Schmitz-Koep et al., 2021). We observed that while the correlation did not differ significantly between the VP/VLBW group and the FT controls, it approached statistical significance towards stronger correlation in the VP/VLBW group, possibly suggesting that related whole amygdala development across hemispheres could be affected by prematurity. In the present study, we investigated structural covariance within the amygdala. We found significantly increased correlation between parts of left BLA in the prematurity group, supporting the hypothesis that development of BLA might be particularly affected by premature birth. Moreover, we found increased structural covariance between subregions of the amygdala, namely BLA, SFA, and CMA, after premature birth. Increased structural covariance has previously been reported after premature birth between grey matter regions including cortical regions, caudate, thalamus and cerebellum and may reflect potential neuroplastic compensatory mechanisms or differences in structural and functional connectivity (Nosarti et al., 2011, Scheinost et al., 2017).

In conclusion, we found decreased BLA nuclei volumes and altered structural covariance within the amygdala. It follows that prematurity has complex effects on amygdala nuclei development persisting into adulthood, particularly for BLA nuclei. Our data support the hypothesis that the BLA is particularly affected by premature birth, possibly due to its developmental dependency on SPNs.

### 4.2 Increased social anxiety is not associated with reduced amygdala volumes in premature-born adults

While we previously found significantly increased avoidant personality T scores in VP/VLBW individuals compared to FT controls reflecting increased social anxiety trait, this trait was not correlated with whole amygdala volume alterations (Schmitz-Koep et al., 2021). In general, studies linking anxiety or personal traits to amygdala volume provide heterogenous results, both in healthy subjects and in patients with anxiety disorders (Avinun et al., 2020, De Bellis et al., 2000; J. C. Gray et al., 2018, Hayano et al., 2009, Qin et al., 2014, Schienle et al., 2011, Spampinato et al., 2009). Animal studies provide ample evidence for functional specializations and parallel processing within amygdala subdivisions (Balleine and Killcross, 2006, Janak and Tye, 2015). Previous morphometric studies in humans (including premature-born populations) did often not differentiate amygdala nuclei, leaving the possibility that differential changes in amygdalar subcircuits remained undetected. Therefore, in the present study, we investigated whether volumetric differences in distinct amygdala nuclei are linked with social anxiety. We did not find an association between specifically reduced volumes of BLA and social anxiety. Maybe, structural changes of amygdalar circuits, as measured by volume changes, do not directly translate into behavioral deficits, but are mediated by changes in functional connectivity (Baur et al., 2013, Hahn et al., 2011, Jalbrzikowski et al., 2017, Johns et al., 2019): For example, in preterm-born adolescents, social impairments were associated with functional connectivity of the amygdala, supporting a possible relationship between prematurity, social anxiety and the amygdala (Johns et al., 2019). Furthermore, multiple brain systems are involved in the mediation of anxiety and social anxiety behavior, such as other regions of the limbic system and prefrontal cortex (J. A. Gray, 1982, Martin et al., 2009, Spampinato et al., 2009, Wu et al., 1991).

In conclusion, neural correlates of social anxiety after premature birth remain less clear and further investigations including other brain regions as well as other structural and functional measures are necessary.

### 4.3 Strengths and limitations

One of the strengths of our study is that a relevant impact of patient age on amygdala volumes at the time of the MRI scan is excluded as VP/VLBW subjects and FT controls had the same mean age of 26 years and age range was very limited. Another strength of our study is a large sample size (101 VP/VLBW individuals and 108 FT controls) which enhances the generalizability of our findings. Third, segmentation quality was improved as both high-resolution T1-weighted and T2-weighted images were used for amygdala segmentation.

However, one important limitation of this study is that amygdala segmentation is challenging due to small regional volumes and limited availability of a clear ground truth. While reliability of the segmentation method used has been investigated and mostly allows for reliable parcellation of amygdala nuclei, validity of segmentation remains unclear. To address this limitation, we first reviewed general reliability of the applied parcellation scheme in previous studies. While Armio et al. (2020) reported excellent test-retest reliability of this segmentation method, Buser et al. (2020) found that medial and paralaminar nucleus showed poor numerical and/or spatial reliability. Therefore, we decided to exclude both medial and paralaminar nucleus from our analyses. Relatively small standard errors and narrow 95% confidence intervals, presented in Table 2 and Table S1, indicate that uncertainty in the estimation of amygdala nuclei volumes is relatively low, and that the sample mean of our data is likely to be close to the ‘true’ population mean.

Another limitation is that individuals with more birth complications in the initial Bavarian Longitudinal Study sample were more likely to be excluded in the initial screening for MRI due to exclusion criteria for MRI. Therefore, the current sample is biased to VP/VLBW adults with less severe neonatal complication and the observed differences in amygdala volumes between VP/VLBW subjects and FT controls are conservative estimates of true differences. However, as mean GA and BW were not significantly different in VP/VLBW subjects with MRI data compared to subjects without MRI data (see Table S3), the sample with MRI data was still representative of the full cohort in terms of GA and BW. There were few subjects with intraventricular hemorrhage in the neonatal period (see Table S4). To investigate whether removing subjects with intraventricular hemorrhage impacts the results, we repeated general linear model analyses for left and right accessory basal nucleus (see Table S5) between the remaining subjects of the VP/VLBW group and the FT group. These results indicate that our main findings of amygdala nuclei volume reductions were not affected by effects of intraventricular hemorrhage.

## 5 Conclusions

Basolateral amygdala seems to be specifically impaired after premature birth, possibly due to disturbance of its distinct claustral developmental pathway. The present study might motivate further investigations of brain systems with subplate-dependent development, particularly in relation to the BLA such as claustrum and insula. Furthermore, future studies should investigate further neural correlates of social anxiety after premature birth including other brain regions as well as other structural and functional measures of the amygdala nuclei. In summary, results suggest lasting and distinct effects of prematurity on amygdala nuclei and their development.

## CRediT authorship contribution statement

**Benita Schmitz-Koep:** Formal analysis, Data curation, Writing - original draft, Visualization, Funding acquisition. **Juliana Zimmermann:** Investigation, Data curation, Writing - review & editing. **Aurore Menegaux:** Investigation, Data curation, Writing - review & editing. **Rachel Nuttall:** Investigation, Data curation, Writing - review & editing. **Josef G. Bäuml:** Investigation, Data curation. **Sebastian C. Schneider:** Investigation, Data curation. **Marcel Daamen:** Investigation, Data curation, Writing - review & editing. **Henning Boecker:** Resources, Supervision. **Claus Zimmer:** Resources, Supervision. **Dieter Wolke:** Resources, Writing - review & editing, Supervision, Project administration, Funding acquisition. **Peter Bartmann:** Resources, Writing - review & editing, Supervision, Project administration, Funding acquisition. **Dennis M. Hedderich:** Conceptualization, Methodology, Writing - review & editing, Supervision, Funding acquisition. **Christian Sorg:** Conceptualization, Methodology, Writing - review & editing, Supervision, Funding acquisition.

## Declaration of Competing Interest

The authors declare that they have no known competing financial interests or personal relationships that could have appeared to influence the work reported in this paper.
