# Supplementary material for: Within amygdala: Basolateral parts are selectively impaired in premature-born adults
Source: Neuroimage Clin. 2021 Aug 9;31:102780. doi: 10.1016/j.nicl.2021.102780 (PMC8374486; doi:10.1016/j.nicl.2021.102780)
Supplement: Supplementary data 1 [file mmc1.docx]

**- Supplement -**

**Title:** Within amygdala: basolateral parts are selectively impaired in premature-born adults

**Authors and Affiliations:**

Benita Schmitz-Koep, MD ^1,2^; Juliana Zimmermann, MSc ^1,2^; Aurore Menegaux, PhD ^1,2^; Rachel Nuttall, MSc ^1,2^; Josef G. Bäuml, PhD ^1,2^; Sebastian C. Schneider, BSc ^1,2^; Marcel Daamen, Dr. phil. ^3,4^; Henning Boecker, MD ^3^; Claus Zimmer, MD ^1,2^; Dieter Wolke, PhD ^5,6^; Peter Bartmann, MD PhD ^4^; Dennis M. Hedderich, MD, MHBA ^1,2^*; Christian Sorg, MD ^1,2,7^*

^1^Department of Diagnostic and Interventional Neuroradiology, School of Medicine, Technical University of Munich, Ismaninger Str. 22, 81675 Munich, Germany;

^2^TUM-NIC Neuroimaging Center, School of Medicine, Technical University of Munich, Ismaninger Str. 22, 81675 Munich, Germany;

^3^Functional Neuroimaging Group, Department of Diagnostic and Interventional Radiology, University Hospital Bonn, Venusberg-Campus 1, Bonn, Germany;

^4^Department of Neonatology, University Hospital Bonn, Venusberg-Campus 1, Bonn, Germany;

^5^Department of Psychology, University of Warwick, University Road, Coventry, CV4 7AL, United Kingdom;

^6^Warwick Medical School, University of Warwick, University Road, Coventry, CV4 7AL, United Kingdom;

^7^Department of Psychiatry, School of Medicine, Technical University of Munich, Ismaninger Str. 22, 81675 Munich, Germany.

*These authors contributed equally to this work.

**Supplementary Tables**

**Table S1: Raw amygdala nuclei volumes**

|  | **VP/VLBW (n = 101)** | | **FT (n = 108)** | |
| --- | --- | --- | --- | --- |
|  | **mean** | **SE** | **mean** | **SE** |
| **Left**  La  Ba  AB  AAA  Ce  Co  CAT  **Right**  La  Ba  AB  AAA  Ce  Co  CAT | 1513.24  554.86  385.61  225.37  50.86  38.87  23.69  167.98  1564.48  591.27  395.96  226.22  54.86  45.64  22.67  161.35 | 21.55  8.05  5.53  3.52  0.85  0.90  0.49  2.71  22.91  8.94  5.88  3.62  0.93  1.13  0.52  2.48 | 1689.66  610.45  429.64  257.75  55.60  44.79  27.60  189.53  1757.03  653.93  442.99  260.69  61.47  54.47  26.89  182.15 | 17.32  6.69  4.61  2.79  0.77  0.88  0.38  2.12  19.24  7.41  5.06  3.09  0.86  1.06  0.46  2.17 |

Raw whole amygdala and amygdala nuclei volumes (in mm^3^) with SE (in mm^3^) in VP/VLBW subjects and in FT controls.

Abbreviations: AAA, anterior amygdaloid area; AB, accessory basal nucleus; Ba, basal nucleus; CAT, corticoamygdaloid transition area; Ce, central nucleus; Co, cortical nucleus; FT, full term; La, lateral nucleus; SE, standard error; VP/VLWB, very preterm and/or very low birth weight.

**Table S2: Group comparison of amygdala nuclei volumes without whole amygdala volume as covariate**

|  | **VP/VLBW (n = 101)** | | | | **FT (n = 108)** | | | | **p-value** |
| --- | --- | --- | --- | --- | --- | --- | --- | --- | --- |
|  | **mean** | **SE** | **95% CI** | | **mean** | **SE** | **95% CI** | |  |
| **Left**  La  Ba  AB  AAA  Ce  Co  CAT  **Right**  La  Ba  AB  AAA  Ce  Co  CAT | 566.80  393.30  230.07  51.71  39.80  24.15  171.26  609.06  407.42  232.26  56.07  47.37  23.12  164.82 | 6.26  4.33  2.80  0.77  0.89  0.44  2.23  6.36  4.47  2.94  0.87  1.04  0.49  2.10 | 554.46  384.77  224.54  50.19  38.04  23.29  166.87  596.51  398.61  226.37  54.36  45.33  22.15  160.67 | 579.14  401.83  235.59  53.24  41.56  25.01  175.648  621.60  416.23  237.95  57.78  49.42  24.09  168.97 | 599.28  422.45  253.36  54.81  43.92  27.18  186.47  637.30  432.27  255.15  60.34  52.85  26.47  178.90 | 6.04  4.17  2.70  0.75  0.86  0.42  2.15  6.14  4.31  2.83  0.84  1.00  0.47  2.03 | 587.38  414.22  248.03  53.34  42.22  26.35  182.24  625.20  423.78  249.56  58.69  50.88  25.53  174.90 | 611.18  430.67  258.69  56.280  45.62  28.01  190.70  649.39  440.77  260.73  61.99  54.82  27.40  182.90 | **<0.001**  **<0.001**  **<0.001**  **0.006**  **0.002**  **<0.001<0.001**  **0.002**  **<0.001**  **<0.001**  **0.001**  **<0.001**  **<0.001**  **<0.001** |

Estimated marginal means of amygdala nuclei volumes (in mm^3^) with SE (in mm^3^) and 95% CI (in mm^3^) in VP/VLBW subjects and in FT controls. General linear models with group membership as fixed factor and TIV, sex and scanner as covariates. Bold letters indicate statistical significance after FDR correction using the Benjamini-Hochberg procedure.

Abbreviations: AAA, anterior amygdaloid area; AB, accessory basal nucleus; Ba, basal nucleus; CAT, corticoamygdaloid transition area; Ce, central nucleus; CI, confidence interval; Co, cortical nucleus; FT, full term; La, lateral nucleus; SE, standard error; VP/VLWB, very preterm and/or very low birth weight.

**Table S3: Comparison between VP/VLBW subjects with MRI data and without MRI data**

|  | **VP/VLBW with MRI (n=101)** | | **VP/VLBW without MRI (n=159)** | |  |
| --- | --- | --- | --- | --- | --- |
|  | **Mean** | **SD** | **Mean** | **SD** | **p value** |
| **GA (weeks)** | 30.5 | ± 2.1 | 30.6 | ± 2.3 | 0.656 |
| **BW (g)** | 1324 | ± 313 | 1323 | ± 320 | 0.980 |

Statistical comparisons: Two sample t-tests. Statistical significance was defined as p<0.05.

Abbreviations: BW, birth weight; GA, gestational age; INTI, intensity of neonatal treatment index; IQ, intelligence quotient; SD, standard deviation; MRI, magnetic resonance imaging; VP/VLBW, very preterm and/or very low birth weight.

**Table S4: Intraventricular hemorrhage**

|  | **VP/VLBW (n=101)** | |
| --- | --- | --- |
|  | **n** | **%** |
| **Intraventricular hemorrhage**  **None**  **Stage 1**  **Stage 2**  **Stage 3**  **Stage 4** | 85  5  7  3  1 | 84.2  5.0  6.9  3.0  1.0 |

Number of VP/VLBW subjects with intraventricular hemorrhage and its grade assessed with ultrasound examinations in the neonatal period, graded 1-4.

Abbreviations: MRI, magnetic resonance imaging; VP/VLBW, very preterm and/or very low birth weight.

**Table S5: Group comparison of volumes of left and right accessory basal nucleus between VP/VLBW subjects without intraventricular hemorrhage and FT controls**

|  | **VP/VLBW (n = 85)** | | | | **FT (n = 108)** | | | | **p-value** |
| --- | --- | --- | --- | --- | --- | --- | --- | --- | --- |
|  | **mean** | **SE** | **95% CI** | | **mean** | **SE** | **95% CI** | |  |
| Left  Right | 235.33  237.07 | 2.86  3.06 | 229.70  231.03 | 240.97  243.11 | 253.52  255.37 | 2.51  2.69 | 248.58  250.07 | 258.47  260.68 | **<0.001**  **<0.001** |

Estimated marginal means of left and right accessory basal nucleus volume (in mm^3^) with SE (in mm^3^) and 95% CI (in mm^3^) in VP/VLBW subjects without intraventricular hemorrhage and in FT controls. General linear models with group membership as fixed factor and whole amygdala volume, sex and scanner as covariates. Bold letters indicate statistical significance.

Abbreviations: CI, confidence interval; FT, full term; SE, standard error; VP/VLWB, very preterm and/or very low birth weight.
